# Editorial: Mental health of children and adolescents of minority groups

**DOI:** 10.3389/fpsyt.2024.1391368

**Published:** 2024-03-15

**Authors:** Yuan Yuan Wang, Yasodha Rohanachandra, Dulangi Dahanayake

**Affiliations:** ^1^ Key Laboratory of Brain, Cognition and Education Sciences, Ministry of Education, Guangzhou, China; ^2^ School of Psychology, Center for Studies of Psychological Application, and Guangdong Key Laboratory of Mental Health and Cognitive Science, South China Normal University, Guangzhou, China; ^3^ Child and Youth Mental Health Services, Latrobe Regional Health, Traralgon, VIC, Australia; ^4^ Child and Youth Mental Health Service, Darling Downs Hospital and Health Service, Toowoomba, QLD, Australia

**Keywords:** mental health, minority, children, adolescents, minority groups

Decades of research has shown that adolescents and youth from minority groups have a higher risk of developing mental health problems (1). Adolescence and young adulthood are a period where individuals expand their social networks, develop their identity and gain insight into their position within society (2). This makes adolescents and youth vulnerable to perceive discrimination, which hinders healthy development and may make them more vulnerable to mental health problems.

In addition to having a higher risk of mental disorders, adolescents and youth from minorities are less likely to use mental health services (3). Mental health stigma among minorities, lack of insurance, lack of culturally competent care providers and language barriers have all been described as potential barriers for young persons from minorities to seek mental health services (4). Furthermore, behavioural concerns of minority youth are more likely to be referred to the juvenile justice system, than to mental health services, which makes them less likely to receive mental health care (4).

In this Research Topic, we describe the mental health of youth from different types of minority groups. Through this Research Topic, we hope to bring to light the recent literature on the mental health of adolescents and young persons from minorities and how to cater mental health services to the special needs of this population.

In terms of ethnic minorities, researchers from China paid attention to the association and mechanism between childhood trauma and mental health among Zhuang adolescents. Yin et al. found that childhood trauma was positively associated with depressive symptoms, in which expressive suppression not only moderated, but also mediated the relationship, particularly in the case of emotional abuse. Wei et al. considered the subtype and number of childhood trauma and found that emotional abuse was the strongest predictor of insomnia and psychotic-like experiences. With respect to children and adolescents with disabilities, Yang et al. highlighted the importance of physical activity in quality of life. Specifically, physical activity positively related to quality of life via self-concept, in which self-concept fully mediated the association. Corresponding mental health services and screening are also essential for this group with a high risk of mental problems. Afshari et al. introduced a 5-hour workshop for teachers, which increased teachers’ knowledge of common mental disorders in school-age children for the detection of psychological problems. This study supports the effectiveness of teacher empowerment training in screening, guiding, and referring schoolchildren to behavioural and mental health problems.

Huang et al. explored the influence of school context on ethnic and racial identity (ERI) development and associated psychological outcomes in ethnic minority adolescents in a longitudinal study in Western North America. The most significant relationships were found for monoracial adolescents, with the school environment significantly predicting ERI and later depressive features. There findings indicated that peer relationships were the most important predictor for the development of depressive features in multiracial youth, highlighting differences from their monoracial peers. Lu et al. studied the occurrence of psychotic-like experiences (PLEs) and their correlations with sexual orientation among college students. Their findings revealed that non-heterosexual college students exhibited a higher risk of PLEs with stratified analysis revealing a significant association for female students. In another study exploring mental health issues among sexual minorities, Huang et al. (5) found that young men from sexual minority groups had higher levels of reported suicidal ideation and lower social support than their heterosexual counterparts. They identified enhancing social support and self-esteem as possible psychosocial interventions to reduce suicidality in men belonging to sexual minorities. In their perspective article on clinical high risk for psychosis in Africa, Awhangansi et al. draw attention to the need for further research in this area in the African setting, development of culturally valid assessment tools and reducing stigma to enhance the utilization of services.

The articles presented in this Research Topic, although diverse, are a testament to the mental health issues affecting minority groups and have the potential to serve as a platform for future studies to identify, manage and prevent such issues in these populations.

## Author contributions

YW: Writing – review & editing. YR: Writing – review & editing. DD: Writing – review & editing.
